# The COVID-19/Tuberculosis Syndemic and Potential Antibody Therapy for TB Based on the Lessons Learnt From the Pandemic

**DOI:** 10.3389/fimmu.2022.833715

**Published:** 2022-02-15

**Authors:** Sylvia Annabel Dass, Venugopal Balakrishnan, Norsyahida Arifin, Crystale Siew Ying Lim, Fazlina Nordin, Gee Jun Tye

**Affiliations:** ^1^ Institute for Research in Molecular Medicine (INFORMM), Universiti Sains Malaysia, Minden, Malaysia; ^2^ Department of Biotechnology, Faculty of Applied Sciences, UCSI University, Kuala Lumpur, Malaysia; ^3^ Tissue Engineering Centre (TEC), Universiti Kebangsaan Malaysia Medical Centre (UKMMC), Kuala Lumpur, Malaysia

**Keywords:** COVID-19, tuberculosis, COVID-19/TB syndemic, therapeutic antibody, T cell receptor (TCR)-like antibody

## Abstract

2020 will be marked in history for the dreadful implications of the COVID-19 pandemic that shook the world globally. The pandemic has reshaped the normality of life and affected mankind in the aspects of mental and physical health, financial, economy, growth, and development. The focus shift to COVID-19 has indirectly impacted an existing air-borne disease, Tuberculosis. In addition to the decrease in TB diagnosis, the emergence of the TB/COVID-19 syndemic and its serious implications (possible reactivation of latent TB post-COVID-19, aggravation of an existing active TB condition, or escalation of the severity of a COVID-19 during TB-COVID-19 coinfection), serve as primary reasons to equally prioritize TB. On a different note, the valuable lessons learnt for the COVID-19 pandemic provide useful knowledge for enhancing TB diagnostics and therapeutics. In this review, the crucial need to focus on TB amid the COVID-19 pandemic has been discussed. Besides, a general comparison between COVID-19 and TB in the aspects of pathogenesis, diagnostics, symptoms, and treatment options with importance given to antibody therapy were presented. Lastly, the lessons learnt from the COVID-19 pandemic and how it is applicable to enhance the antibody-based immunotherapy for TB have been presented.

## Introduction

The coronavirus (COVID-19) pandemic is an ongoing deadly viral infection, affecting globally to date. Initially reported at Wuhan City, China, in December 2019, the highly contagious airborne virus has spread all over the world, infecting approximately 199 million individuals with a staggering death toll of almost 4.3 million people worldwide (as of 5^th^ August 2021) (1). The dreadful consequences of the pandemic have affected the normality and quality of human life, economy and financial stability (2), mental and physical health (especially front line workers) (3), and sadly adolescent growth and development (4). While the world battles and focuses on the COVID-19 pandemic, it is also imperative not to lose focus on another air-borne disease, Tuberculosis (TB). Since the pandemic started, the World Health Organization (WHO) has reported an alarming reduction in the TB cases diagnosed and patient care worldwide in 2020, especially from the high TB-burden countries (28% reduction as compared to 2019) which may consequently increase the TB death toll to an added 0.5 million deaths (5, 6). The primary contributing factor to this scenario is associated with the shift of resources such as the healthcare workforce, monetary and diagnostic instruments (GeneXpert), from TB to COVID-19 pandemic (7, 8).

Another important reason to focus on TB during this pandemic is the emergence and potential implications of the COVID-TB cursed-duet/syndemic. In this scenario, the synergistic interaction between COVID and TB can further aggregate the burden of the disease and subsequently impact the health quality within a population (9). Prior to COVID, the HIV-TB was and still is a well-evident syndemic reported in different parts of the world, affecting both adults and children, and needless to say stand as a major obstacle for the elimination of TB disease worldwide (10, 11). Overall, the COVID-TB syndemic has several possible implications which include the reactivation of latent *Mtb* after SARS-CoV-2 infection, COVID-TB co-infection which consequently may lead to the aggravation of an existing active TB condition or an existing *Mtb* infection may escalate the risk and severity of SARS-CoV-2 infection (12).

The reactivation of latent tuberculosis in post-COVID infected patients has been one of the most concerning setbacks of the COVID-TB syndemic. This scenario differs from a COVID/TB co-infection in which a patient experiences both COVID-19 and TB simultaneously. One possible factor for this progression is the CD4^+^ T cells which are the key immune defenders against mycobacterium tuberculosis (*Mtb*) but unfortunately found to be exhausted and reduced in COVID-19 patients (13). According to the case report by Elziny and colleague, a 29-year old healthy male (no serious illness) from Qatar with no prior history or exposure to *Mtb*, was diagnosed with miliary pulmonary TB two weeks after recovering from COVID-19 infection (14). The patient’s latent tuberculosis status was not stated although initial findings suggested peritoneal tuberculosis or pseudomyxoma peritonei (Acid-fast bacilli, PCR, and cytology test from peritoneal fluid tapping was negative). Hence, a possible reactivation of latent tuberculosis due to COVID-19 cannot be concluded. More reliable evidence of latent TB reactivation from COVID-19 was reported in a 40-year old female with possible latent TB, who developed active tuberculosis 7 weeks after her initial infection with COVID-19 (15). With one-fourth of the global population being affected with latent tuberculosis (16), it is essential to constantly be aware of the serious effects of COVID-19, especially during and post-infection in potentially activating latent tuberculosis in COVID patients and perhaps implements a standard simultaneous COVID-19 and latent TB validation test to ensure effective treatment is given.

**Figure 1:**
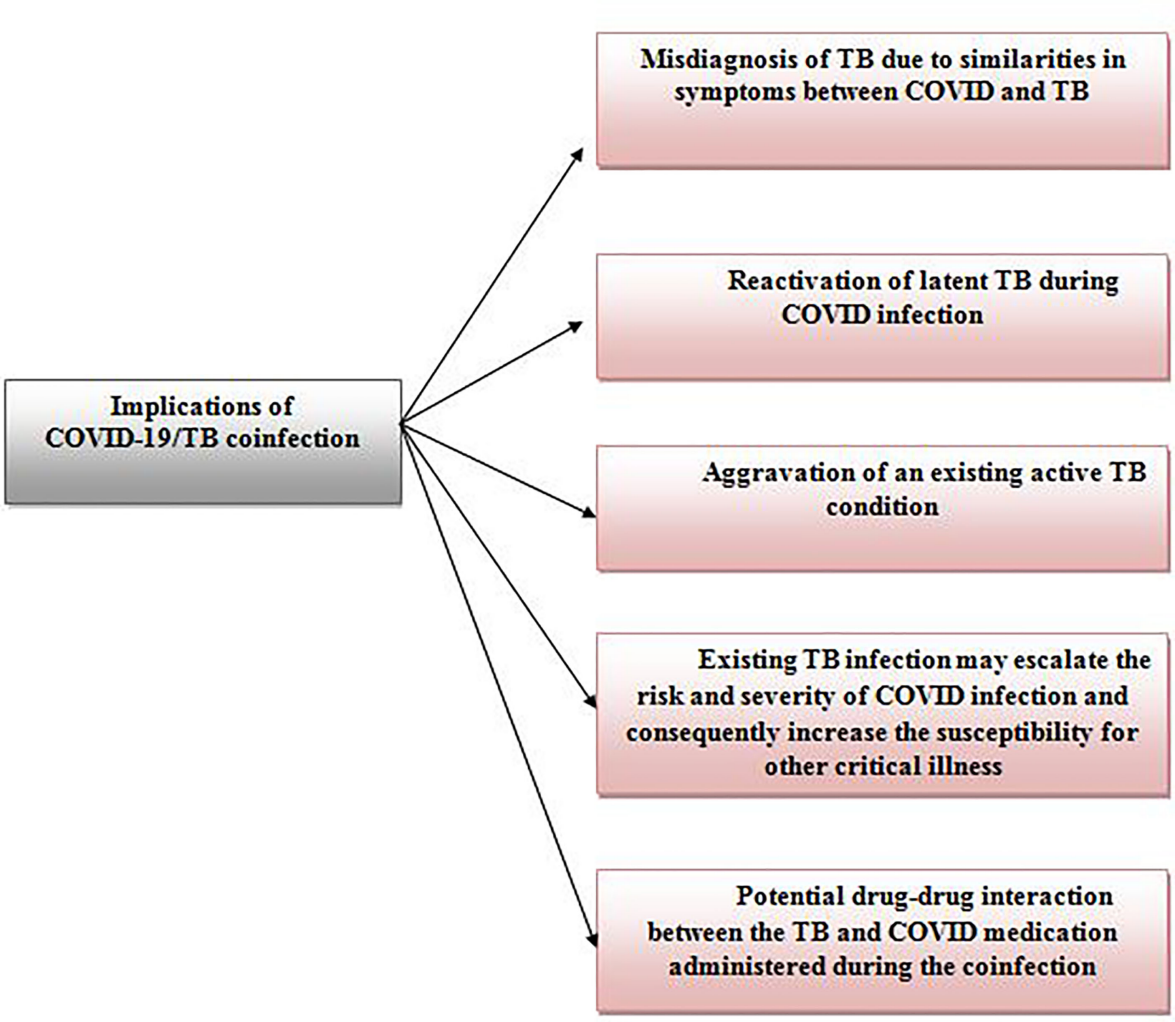
The implications from the co-infection of COVID-19 and Tuberculosis.

The co-infection of COVID-19 and TB is another prominent implication of the COVID-TB syndemic, recorded worldwide to date. TB co-infection is not a novel phenomenon as substantial records of TB co-infection has been reported in the past with diseases such as human immunodeficiency virus (HIV) (17), malaria (18), Middle East respiratory syndrome (MERS) (19), and severe acute respiratory syndrome (SARS) (20). However, COVID/TB co-infection is notably alarming as it can result in serious implications (12, 21) as shown in **Figure 1**. The possible mechanisms contributing to the Covid/TB co-infection have been well elaborated by Mousquer et al. (12). The first incident on COVID-19/TB co-infection was reported in China where 3 patients with a past history of tuberculosis (2 patients: pulmonary TB and 1 patient: untreated TB) were diagnosed with COVID-19 and TB *via* real-time polymerase chain reaction (RT-PCR) (22). Since then, COVID-19/TB co-infection has been recorded in various countries including India (23), Mexico (24), Saudi Arabia (25), Italy, South Africa and, the Philippines (12), with a range of good (26) to poor prognosis (fatality) (27).

Overall, the evidence reported so far emphasizes the urgent need to focus on tuberculosis as much as COVID-19 from various perspectives to ensure efficient treatments are given and ultimately resolve the catastrophic effects of the COVID-19/TB syndemic. In this review, we have discussed the crucial need to focus on TB in the midst of COVID-19 pandemic. Besides, a general comparison between COVID-19 and TB in the aspects of pathogenesis, diagnostics, symptoms, and treatment options with importance given to antibody therapy were presented. Lastly, the lessons learnt from the COVID-19 pandemic and how it is applicable to enhance the antibody-based immunotherapy for TB has been presented.

## COVID-19 and Tuberculosis

It is an undeniable fact that a parallel line can be observed between COVID-19 and TB although both diseases vary from each other.

### Pathogenesis

In terms of pathogenesis, both Tuberculosis and COVID-19 have different causative agents with *Mycobacterium tuberculosis* complex (*Mtb*) and severe acute respiratory syndrome coronavirus 2 (SARS-CoV-2) responsible for TB (28) and COVID-19 (29) infection respectively. Being airborne diseases, both diseases share a similar transmission pathway in which the pathogen is transmitted from an infected individual *via* respiratory fluid droplets or aerosol in the event of sneezing, coughing, or talking (30). However, SARS-CoV-2 can also be transmitted by having direct contact with a virus-exposed surface and subsequently touching different parts of the face especially the nose (31). This is the primary reason hand sanitization is recommended as one of the preventive measures for COVID-19 and is strongly implemented as a standard operating procedure in different countries. Both *Mtb* and SARs-CoV-2 target primarily the respiratory system (32, 33). There is however a difference in the infection process since the virus (requires a host for replication) and bacteria undergo different replication approaches. Upon inhalation, the SARS-CoV-2 virus utilizes the epithelial cells as hosts and initiates infection by binding to the angiotensin-converting enzyme 2 (ACE2) receptors of the host (34). The ACE2 receptors are found to be expressed in the epithelial cells of many human organs and are known to contribute to the multiple organ failure experienced by COVID-19 patients in the critical stage (35). An in-depth explanation of the role of ACE2 in the pathogenesis of COVID-19 has been well discussed by Ni et al. (36). On the other hand, the inhaled *Mtb* migrates through the respiratory tract and ultimately reaches the alveoli of the lungs (32). There, the bacteria are subjected to phagocytosis by the innate immune defense cells including alveolar macrophages and dendritic cells resulting in two distinctive possible scenarios (37). The first possibility is the activation of pro-inflammatory immune responses and activation of CD4+ and CD8+ cells to confine the infection from spreading and ultimately eliminate the infected antigen-presenting cells (38). The second possibility occurs in the event of *Mtb* overcoming the immune defenses (active tuberculosis). The *Mtb* bacilli engulfed by the alveolar macrophages, survive the defense mechanism and replicate in the macrophage leading up to its necrosis (39). This allows the surviving bacilli to replicate extracellularly and spread to other parts of the body besides the lungs through the lymphatic and blood system (40). In latent tuberculosis, *Mtb* bacilli are able to evade the intense host immune defenses, survive the stressful microenvironment and progress to a dormancy state while resisting the eradication from the immune system (41). The bacilli remain dormant until there are opportunities for reactivation which in most cases are due to immunosuppression or weaken immune system due to infections including HIV and COVID-19, malnutrition, tobacco smoke, air pollution, alcoholism, diabetes, kidney failure and malignancy (15, 41, 42).

### Symptoms

The similarities in the majority of the symptoms tie COVID-19 and TB in a negative aspect especially in the scenario of co-morbidity. Some of the shared symptoms such as cough, fever, lethargy, loss of appetite and shortness of breath, masks the diagnosis of TB during COVID-19 and vice versa consequently delay/effects the treatment process that a patient deserves (43). Unfortunately, this has the potential and in one case, led to fatality (27, 44). One way to prevent this scenario is to practice a standard dual diagnostic testing for TB (latent and active) and COVID-19 simultaneously without weighing in on the costing as this will enhance the overall treatment efficacy and minimize the uneventful effects of TB/COVID syndemic. TB can be distinguished from COVID-19 with additional symptoms such as weight loss, night sweating, and blood in the sputum (45) while COVID-19 can be differentiated from TB with symptoms like loss of smelling and tasting sense, headache, sore throat, body ache, congestion, diarrhea, and even vomiting (46).

### Diagnostics

Before the COVID-19 pandemic, the diagnosis of TB is established by tuberculin skin test, blood test, microscopic evaluation of patient samples (sputum, bronchoalveolar lavage fluid, a biopsy sample and, etc), imaging, and advanced molecular testing such as nucleic acid amplification testing (NAATs) (47, 48). However, the lessons learnt from the COVID-19 pandemic has remodeled the approaches for TB diagnosis in terms of providing easy access for TB testing through drive-in and mobile testing which can be implemented in many places convenient to the public people, self-diagnosis TB kit, replicating the artificial intelligence technology used in analyzing x-ray images of COVID-19 patients and concurrently apply for TB diagnosis and lastly explore molecular technologies with enabling the diagnosis of multiple diseases such as COVID-19, HIV and TB in a single approach (49). As for COVID-19, real-time reverse transcription-polymerase chain reaction (RT-PCR) remains the golden standard to detect the viral gene of the SARS-CoV-2 virus in patient samples (50). Other alternatives include antibody and antigen detection methods *via* lateral flow (LAF) and enzyme-linked immunosorbent assay (ELISA) and even rapid antigen testing using nasal samples or saliva (self-test) which has made COVID-19 testing easier to accommodate large routine testing in workplaces and standard testing prior to attending public events (51).

### Treatment

The standard treatment for TB as per the WHO guidelines include different anti-microbial drug regimens of rifampin (52), isoniazid (53), and isoniazid plus rifapentine (54), administered for a specific duration depending on the suitability to the patient (55). In the event where a patient is diagnosed with multidrug-resistant TB (MDTB) or extensively drug-resistant TB (XDR TB), secondary drugs such as thioamides, ethambutol, cyclic peptides, etc for MDTB and bedaquiline, delamanid, ethambutol, etc for XDR TB will be administered respectively (56). Multidrug-resistant TB strains exist as a result of specific mutations in *Mtb* which ultimately reduced the efficacy of the anti-microbial drug. For instance, mutation of catalase-peroxidase KatG and promoter region *inhA* is associated the inefficiency of anti-TB drug, isoniazid (57). Meanwhile, the resistance to rifampicin is a result of the mutation of rpoB in *Mtb* (58). The in-depth discussions on how each mutation affects the efficacy of anti-TB drugs was well-elaborated in the past (59–62).

Since COVID-19 is a relatively novel disease with varying severity, a standard treatment regimen is not applicable rather COVID-19 patients treatment is planned based on the disease severity and the clinical symptoms exhibited. For non-symptomatic to mild COVID patients, self-isolation for 14 days is recommended along with basic cough, runny nose, fever and even pain reliever medication (if necessary) and proper hydration (63, 64). Patients in moderate to severe stages require hospitalization and require different therapeutic approaches including antiviral therapy, monoclonal antibody therapy, anti-inflammatory drugs and symptom-specific medications (65). The first U.S. Food and Drug Administration (FDA) approved COVID-19 antiviral drug was remdesivir, a viral RNA-dependent inhibitor that inhibits viral replication of the SARS-CoV-2 virus *in vitro* (66, 67). In addition, emergency use authorization (EUA) has also been granted to monoclonal antibodies, bamlanivimab and REGN-COV_2_ (casirivimab and imdevimab), to be incorporated in COVID-19 therapeutics (68). Similar to TB, the mutations of the SARS-CoV-2 virus not only led to the emergence of the highly infectious Delta and even severe Omicron strains, but also significantly reduce the efficacy of the available treatment options for COVID-19 (69). The Omicron strain has more than 30 mutations which contributes to its transmissibility and ability to overcome the anti-viral drug and several monoclonal antibodies (70). Further investigation is necessary to understand the mechanisms elicited by these mutations and ultimately generate strategies to combat Omicron.

### Vaccination

The history of TB vaccination can be dated back to 1921 when the bacilli Calmette-Guerin (BCG) vaccine which originated from attenuated *Mycobacterium bovis* was first introduced (71). Remaining as the only licensed vaccine for TB to date, the vaccine is expected to provide protection against severe forms of TB (meningeal and miliary) and decrease TB-related fatality (72). Despite being relatively successful in delivering protection against TB in adolescence, BCG still faces major criticism for losing its efficacy against pulmonary TB in adults which is evident through the still high TB statistics reported currently (73). Another setback is the inconsistency in vaccine efficiency described by different countries (74). Nevertheless, BCG remains as the only vaccine for TB and is part of the national immunization program of many developing countries with high TB burden (75). The ongoing research on TB vaccine focuses on both prevention and therapeutic vaccines which include whole-cell, subunit (adjuvant), DNA and RNA-based, and viral vector-based vaccines [Extensively reviewed by (73, 76, 77)]. Besides, it is of no surprise that many vaccine candidates are under clinical trial evaluation with the rapid development in technology (78). Moving forward to 2020, what seems to be unacceptable is the rapid development and approval obtained for COVID-19 vaccines in contrast to the TB vaccine candidates that are still under evaluation despite being existed way longer before COVID-19. The success of the development of the COVID-19 vaccines can be attributed to the emergency state itself resulting in the rapid development largely supported by both public and private financial resources, expanded manufacturing and consequently the emergency authorization is given to cope with the pandemic (79). Learning from this, it is about time that TB vaccine candidates are given equal if not more opportunities, funding, and emergency so that the disease can be tackled more efficiently.

Since the COVID-19 pandemic was declared, researchers have worked tirelessly towards the development of COVID-19 vaccines in efforts to minimize the severity of the disease and hopefully to control disease progression. As mentioned above, the strong financial support from both private and public resources have made COVID-19 vaccine development rapid and very much possible in a short duration (80). In general, the developed and authorized vaccines can be categorized into mRNa-based (Pfizer, Moderna and CureVac), viral vector (adenovirus)-based (Astra Zeneca, CanSino, Johnson & Johnson and Sputnik V), attenuated whole-cell virus (Bharat Biotech, Sinovac and Sinopharm) and protein subunit vaccine (Novavax) (81). The technology, expected immune responses and the efficacy of the available vaccines have been extensively reviewed by many (82–85). The common side effect observed post-vaccination include fever, fatigue, body ache (mainly injection site), headache, nausea and diarrhoea (86). However, several adverse side effects such as thrombosis with thrombocytopenia (87, 88) (Astra Zeneca and Johnson & Johnson) and Guillain-Barré syndrome (Astra Zeneca) (89) were reported in some post-vaccination incidents. Implemented in stages, starting with the healthcare workers, senior citizens, adults and children (only specific age), fully vaccinated status was given upon completing two doses of COVID-19 vaccines (90, 91). With the emergence of the highly infectious Delta and lately Omicron variants along with the reduced vaccine effectiveness observed over time, a booster shot was recommended (92). Most importantly, there are substantial reports that suggest double doses of COVID-19 vaccines are not sufficient to tackle the highly infectious Omicron variant (93, 94) highlighting the importance of vaccination in general and taking the booster shot. The efficacy of the booster shot (Pfizer) was reported in Israel with a 90% lower mortality rate after the booster shot was taken with a 5 months interval to the second dose (95). The effectiveness of the booster shot was also observed against mild to severe COVID-19 infection in England (96). Since then, many countries have implemented booster shots as part of their COVID-19 vaccination program. Overall, continuous monitoring and improvement are crucial despite the success of the developed vaccines in extinguishing the severity of the COVID-19 pandemic to ensure its efficacy and prolonged protection against COVID-19 in the long run.

At the beginning of the pandemic, researchers looked into the possibility of utilizing established treatment options including BCG vaccination to combat COVID-19. The theory was hypothesized based on the non-specific protection delivered by BCG vaccines against various ‘off-target’ infections which led to the question if the BCG vaccine could provide protection against COVID-19 (97). Initially, the opinions were divided with some agreeing to the ability of BCG to decrease the severity of COVID-19 while the others concluded BCG’s failure to offer protection against COVID-19 through clinical trials (98). However, several clinical trials (Phase III) are being conducted to evaluate the effects of BCG vaccination in reducing the severity of COVID-19 among healthcare workers (99). Another phase III trial investigated the safety of BCG vaccination in the elderly group and concluded the safety and efficacy in protecting the elderly group against respiratory infections (100). These findings may provide a fundamental platform to investigate BCG’s efficacy against COVID-19 elderly patients. Another important preclinical study investigated the combined therapeutic potentials of BCG and COVID-19 vaccines against the SARS-CoV-2 virus *in vivo* (101). It would be beneficial for the investigation to be expanded in clinical settings as the outcome would not only provide essential findings on protection against COVID-19 but most importantly on the dual protection during TB-COVID-19 coinfection which is crucial in high TB burden countries. So far, the evidence from several preclinical studies is pointing towards the BCG vaccine’s ability to promote protection against the SARS-CoV-2 virus (102–105). Nevertheless, more evidence especially from a larger setting is needed to provide a stronger platform for this idea to be applicable.

## Antibody Therapy

Monoclonal antibody therapy has added great therapeutic value and enhanced the treatment efficacy for many diseases including cancer (106), infectious disease (107) and immunosuppression (108) in recent years. The credit to this success undeniably goes to the advances in technology such as hybridoma and phage display technology (109). The generated therapeutic monoclonal antibody is able to perform similar effector mechanisms as a typical human antibody which includes neutralization, activation of the complement cascade, antibody-dependent cellular cytotoxicity (ADCC) and antibody-dependent phagocytosis (ADP) (110). Besides, therapeutic antibodies can also function as fusion molecules by tagging an antibody with a specific toxin, antigen or even drug (111). As inhibitors, the antibodies are capable of blocking certain receptors (EGFR and HER2) which consequently inhibit a pathway and prevent disease progression (112, 113).

### Therapeutic Antibody for COVID-19

In terms of therapeutic antibodies for COVID-19, the scenario is far different from other diseases. The dreadful effects of the COVID-19 pandemic have amplified and expedited the research process to develop treatment alternatives including antibody therapy. Another deviation from normal is how fast the FDA approved emergency authorization for use (EAU) for several therapeutic monoclonal antibodies and anti-viral therapy in efforts to reduce the burden of the pandemic.

The first US-FDA-authorized monoclonal antibody for COVID-19 treatment was bamlanivimab/LY-CoV555 (9^th^ November 2020) (114). Originated from the convalescent plasma of COVID-19 patient from the USA, the antibody is found to exhibit neutralization effects against the SARS-COV2 spike glycoprotein by interfering with the interaction between the virus’s receptor-binding domain (RBD) with the host ACE2 receptor which is crucial for viral entry (115). Bamlanivimab is recommended for the treatment of COVID-19 infected adolescents and adults in the mild to the moderate stage and has indicated a significant reduction in the viral load along with reducing disease progression to the severe stage (116). Initially administered as monotherapy, bamlanivamb was efficient in significantly reducing the viral load by Day 11 in outpatients with mild/moderate stage of COVID-19 infection and subsequently decrease the rate of hospitalization as compared to the control group (117). Similar neutralization effects were obtained when bamlanivamb was administered in combination with another neutralizing antibody known as etesevimab and eventually, the two antibody combinations gained EAU from the US FDA to treat mild to moderate COVID-19 adult and pediatric patients (118, 119). Similar to bamlanivamb, etesevimab which originates from the convalescent plasma of a COVID-19 patient from China, is also able to neutralize the SARS-COV2 virus by binding to RBD receptor but in an overlapping manner to bamlanivimab (120, 121). However, the emergence of a novel SARS-COV-2 variant, B.1.617 also known as the Delta variant, has resulted in the re-evaluation of bamlanivamb’s efficiency. The reason being the Delta variant is found to have distinct mutations in the RBD and N-terminal domain resulting in increased replication and infectivity rate (122). In specific, the mutations in the RBD not only increase the binding affinity of RBD to ACE2 receptor but also reduce/inhibit the neutralization effects of monoclonal antibodies including bamlanivamb by preventing antibody recognition and promoting antibody escape (123). Unfortunately, recent findings have validated the inefficiency/reduced efficiency of bamlanivamb against the Delta variant (123, 124). On the other hand, etesivimab is found to maintain its neutralization activity against the Delta variant (123). Based on *in vitro* findings, the combinational antibody therapy of bamlanivamb and etesevimab is expected to maintain the neutralization effects against the delta variant and therefore is authorized for the treatment of mild to moderate COVID-19 patients (125).

Joining the list of authorized monoclonal antibodies for COVID-19 therapeutics is a cocktail of non-competing antibodies (casirivimab and imdevimab) known as REGEN-COV (126). Administered together, the recombinant neutralizing antibody cocktail inhibits the entry of the SARS-COV2 virus to the host cell by binding to the spike protein RBD in a non-overlapping manner (127). REGEN-COV has not only successfully reduced the viral load in COVID-19 mild to moderate patient, but also decrease the need for hospitalization (128). In a more recent study, similar beneficial findings were obtained when the antibody cocktail was administered to high-risk patients (age>65 years old, overweight, chronic disease or immunocompromised) with mild to moderate COVID-19 (128). In addition, the efficacy of REGEN-COV was further proven when it was able to prevent symptom progression to the severe stage as well as hospitalization requirement of mild to moderate COVID-19 solid organ transplant recipients who are considered high-risk (129). Most crucially, the neutralizing activities of both casirivimab and imdevimab against the highly infective Delta variant were proven in a recent finding (123).Unfortunately, recent finding has indicated the reduced efficacy of REGEN-COV against the Omicron variant (130).

In May 2021, the U.S.FDA granted EUA for another antibody with dual-functionality known as sotrovimab to treat mild to moderate COVID-19 and high-risk patients (131). Similar to the other neutralizing antibodies discussed above, sotrovimab interferes with the interaction of the SARS-COV-2 spike protein and ACE2 receptor by binding to a preserved binding site of the spike protein which consequently inhibits membrane fusion upon viral-ACE2 receptor binding (132). Derived from the S309 antibody isolated from SARS-COV (SARS) patient in 2003, sotrovimab has undergone modification to the FC region to increase its half-life and distribution coverage (133). The highlight of this antibody is the ability to mediate dual defense mechanisms of antibody-dependent cellular cytotoxicity (ADCC) and antibody-dependent cellular phagocytosis (ADCP) (134). In addition, the antibody was also found to maintain its defense mechanism against a variety of SARS-COV-2 variants including the Delta and Omicron variant (133, 135, 136).

Although viral neutralization by preventing the binding of the SARS-COV-2 spike protein RBD to the host ACE2 has been the fundamental approach for the development of most COVID-19 therapeutic antibodies, researchers are also looking into other strategies with targets such as cytokines (GM-CSF, IL-6, IL-17A), angiotensin II (Ang II), tumor necrosis factor (TNF), inhibitory immune checkpoints (PD-1), and complement components (C5, C5a, C5aR) (137). To date (as of 1 August 2021), there are approximately 217 different studies on therapeutic antibodies against COVID-19 under different stages and clinical trial phases (138).

### Therapeutic Antibody for Tuberculosis

Despite being in existence for a long time now, there are still no monoclonal antibodies approved for the therapeutics of TB to date. The endemic status of TB itself, whereby the disease is concentrated in several developing countries could be a possible reason for the delay in antibody development. This is a contrast to the drastic efforts taken for therapeutic antibody development and FDA approvals given to combat the COVID-19 pandemic. In terms of research developments, it comes as no surprise that researchers focus on developing a treatment for TB based on cell-mediated immunity (CMI) mainly due to the intracellular nature of the MTB pathogen. However, CMI cannot be solely reliable in treating TB, especially in immunosuppressive scenarios such as HIV and even COVID-19, whereby the dysfunctionality of T cells is observed. In addition, the inconsistency in the outcome of many studies on antibody-mediated immunity (serum therapy) could also be the reason for the shift of interest (139). Besides, factors such as TB infection stage, bacterial expression, co-infection, HLA specificity and effects of TB treatment, can contribute to the generation of TB therapeutic antibodies (140). Nevertheless, the development of science and technology along with recent evidence (**Table 1**) provide some hope and positive direction for the potential applications of therapeutic antibodies for TB in future.

**Table 1:** Potential antibodies for the immunotherapy of tuberculosis.

Antibody	Target Antigen	Approach	Impact	Reference
Serum antibody (Human)	Lipoarabinomannaan (LAM), component of *Mtb* cell wall	*In vitro*	Internalization of BCG Inhibition of mycobacterial growth Enhance cell-mediated immune response	(141)
9d8 (Murine IgG3)	Arabinomannaan	*In vivo*	Alteration of granulomas in the infected lungs Potentially enhance cell-mediated immunity	(142)
Human intravenous immunoglobulin (IVIg)	Virulent *Mtb* (H37Rv)	*In vivo*	Bacterial load reduction	(143)
Human gamma globulins	BacillusIn Calmette-Guerin (BCG)	*In vivo*	Inhibition of BCG colonization in the lungs	(144)
Human gamma globulins	Virulent *Mtb* (H37Rv)	*In vivo*	Prophylactic effect Inhibition of bacteria infectivity	(145)
SMITB14 monoclonal antibody	Virulent *Mtb* (H37Rv)	*In vivo*	Reduction in bacterial load Reduce weight loss Enhance long-term survival	(146)
TBA61 and TBA84 monoclonal antibody	Virulent *Mtb* (H37Rv)	*In vivo*	Reduction in bacterial load Mild morphometric and histological changes	(147)
TBA61 TB68 TBA84 (Intranasal delivery	Virulent MTB (H37Rv)	*In vivo*	Reduction in bacterial load in the lung	(148)
Human serum antibody	Virulent *Mtb* (H37Rv)	*In vivo* *In vitro*	Moderate protection against *Mtb*	(149)
Apa GroEL	Virulent *Mtb* (H37Rv)	*In vitro*	Inhibit bacterial growth Induce antibody dependent cellular and neutrophil phagocytosis	(150)
IgA IgG IgM (human sera)	LAM Rv2031 HBHA	*In vitro*	Variation in responses between the three antibody isotypes	(151)

One of the current strategies in the development of therapeutic antibodies for TB is serum-based therapy (passive transfer). In this approach, the serum that originated from various sources (guinea pig, bovine, donkey and horse) were found to elicit favorable immune responses against MTB in both *in vitro* and *in vivo* settings (152). Although encouraging, a setback observed from these studies was inconsistency in the findings as mentioned above, possibly contributed by variation in the severity of the disease, sample size, and variation in experimental setup and analysis. In recent approaches, the efficiency of passive transfer was tested *in vivo* using hyperimmune serum of *Mtb*-infected animal model (153), human intravenous immunoglobulin (IVIg) derived from *Mtb*-exposed volunteers (143), human secretory immunoglobulin (Ig)A and IgG (154) and even sera of healthcare workers (latent/highly exposed with negative TB diagnosis) (149) which all sera were able to deliver a certain extent of protection against *Mtb*. These favorable findings may serve as stepping stones for broader investigations.

The second strategy of utilizing monoclonal antibodies for TB therapeutics *in vivo* came along with the success in hybridoma and phage display technology. Several mAbs have been subjected for testing against different *Mtb* antigen targets as described in **Table 1** and have demonstrated a wide range of favorable responses including enhanced survival rate, decreased colony-forming unit and bacterial viability, induce the formation of granuloma and mucosal protection *via* Ig A (155). The hiccups in these studies are the specific defense mechanisms mediated by the antibodies along with the antibody’s specificity were not well defined (150). Moving forward, these aspects could be focused to generate valuable inputs for expanding the application of monoclonal antibodies at the clinical level.

In another prominent approach, antibodies and vaccines are used hand in hand to enhance the therapeutic efficacy for TB. Here, the vaccine with specificity to a particular *Mtb* antigen (Ag85b) is first administered followed by passive transfer of the vaccinated mice serum to *Mtb*-induced mice (156). Consequently, prolonged survival and a decrease of bacterial load in the lung were observed, credited to the antibody’s ability to induce transcriptional changes in the bacterium. A similar vaccine-evoked antibody response (purified protein derivative (PPD)-specific humoral response) was also observed in a rhesus macaque model (157). While testing the efficacy of Ag85A recombinant vaccine (Vaccinia Ankara) in phase 2 clinical trial, researchers found that the tested individuals acquired Ag85A-specific IgG leading to a decrease in TB progression although the vaccine on its own did not demonstrate significant efficiency compared to the BCG control (158). Similar to the previously discussed strategies, the role of antibodies in delivering some form of protection against *Mtb* is evident. However, understanding and further investigating the specific mediating roles of antibodies in this combinational approach would further increase its therapeutic value.

Overall, the many encouraging pre-clinical findings of various experimental models have highlighted antibodies’ crucial role in tackling TB despite the skepticism surrounding it. Besides, TB coinfection with HIV and now the TB-COVID-19 syndemic (dysfunctionality of T cells), have taught us to crucially scout for alternative treatment approaches independent of T cell mechanisms. Therefore, it is about time for antibody-based TB therapeutic strategies to be given enough opportunity to move forward. Given the current emergency, there is very much a high possibility for these TB therapeutic antibodies to be authorized for further clinical evaluations and hopefully for use in treatments.

## Lessons Learnt From COVID-19 Pandemic

It is undeniable that the COVID-19 pandemic has reshaped the normality of life and affected us in many aspects. However, the lessons learnt from the pandemic along with the experiences gained will help mankind to shape a better future and provide a stronger platform to be prepared for any dreadful event in time to come. This part of the review focuses on the lessons learnt from the COVID-19 pandemic that will provide useful insights for the development of therapeutic antibodies for Tuberculosis. One of the biggest lessons learnt from the pandemic is the implications of the TB/COVID-19 syndemic. The three major concerns are the reactivation of latent TB during/post-COVID-19 infection, aggravation of an existing active TB condition during TB/COVID-19 co-infection or an existing *Mtb* infection may increase the susceptibility and severity of SARS-CoV-2 infection. There is substantial evidence to support these implications (12, 15, 21, 159–161).

Latent *Mtb* reactivation during/post- SARS-CoV-2 infection is associated with the depletion and exhaustion of T cells. In severe COVID-19 conditions, excessive cytokines are released in order to combat the viral infection (also known as cytokine storm) resulting in a hyper inflammation state/syndrome (162). Recent findings have validated that cytokine storm is responsible for the depletion and exhaustion of T cells in COVID-19 patients (13). Since CD4 T cells are crucial key players in the immune defense against TB, the exhaustion of T cells in COVID-19 patients can not only contribute to the reactivation of latent TB but also the aggravation of an existing active TB infection during TB-COVID-19 co-morbidity (163–165) Besides, anti-inflammatory drugs such as corticosteroids administered to treat COVID-19 patients have the potential to create an immunosuppressive state which creates opportunities for different infections including reactivation of latent TB to occur (166). On the other hand, the increased susceptibility and severity of COVID-19 by an existing active *Mtb* infection is linked to the elevated circulating myeloid-derived suppressor cells (MDSCs) during active *Mtb* infection (167). There is also a direct correlation between the MDSCs and COVID-19 severity and the cells are known to suppress the responses of T cells to ensure the survival of the virus (168). It is also important to take into account the possibility of the pre-existing lung damages and other health complications implicated by TB in increasing the susceptibility and severity of COVID-19 and vice versa (T cell depletion in COVID-19 patients increase reactivation of latent TB) (169).

The pandemic has taught us to equally view TB from the perspective of the TB/COVID-19 syndemic rather than TB alone. The intersection between all three implications of the TB-COVID-19 syndemic is T cell dysfunctional (depletion, exhaustion or suppression). As such, this important factor should be considered in the development of therapeutic antibodies for TB.

## T Cell Receptor (TCR)-Like/Mimic Antibody

T cell receptor (TCR)-like/mimic antibody is a novel antibody class that has come to the limelight in recent years with the emergence of hybridoma and phage display technology. As the name suggests, the antibody mimics a T cell receptor’s role in identifying/detecting the antigenic peptide presented on the major histocompatibility complex (MHC) molecules class I and subsequently mediates a wide range of effector mechanisms (170). The antibody is an excellent candidate for TB therapeutics especially in the context of TB/COVID-19 syndemic as its effector mechanisms are mostly shielded by T cell exhaustion. The dual-functionality of TCR-like antibody bridges the two arms of the adaptive immune system in the sense that the antibody is able to provide immunosurveillance by detecting the intracellular pathogen of the infected cells (cell-mediated immunity) as well as mediate typical antibody defense mechanisms such as ADCC, ADCP and activation of the complement cascade (humoral antibody) (171).

TCR-like antibodies would be particularly beneficial in latent TB therapeutics. The reason being the antibodies are capable of providing immunosurveillance and subsequently preventing disease progression to active tuberculosis. Unlike a typical antibody that detects soluble or membrane-bound three-dimensional antigen structures, the TCR-like antibodies are capable of mimicking the function of TCR by recognizing dormant TB antigenic peptide presented by the MHC class I molecules of the alveolar macrophage (*Mtb* survives and remains dormant within the alveolar macrophages (172–174). The TCR-like antibodies then can mediate several defense mechanisms including antibody-dependent cellular cytotoxicity (ADCC), opsonization which promotes phagocytosis by phagocytic cells and intracellular eradication *via* Fc receptor-mediated phagocytosis, and activation of the complement system which can potentially lead to the efficient eradication of the dormant TB bacilli (140, 175, 176). Besides, TCR-like antibodies can also have a tremendous impact on immunosuppressed or weakened immune system patients like HIV patients. HIV patients are susceptible to TB as the virus hampers the function of CD4 T cells which contributes significantly to the immune defenses against tuberculosis (38). Additionally, the upregulation of Tregs in TB-infected HIV patients suppresses the effector immune response in the lung, by inhibiting the activation and differentiation of T cells as well as preventing the migration of T cells to the infection site (177). As such, TCR-like antibody is capable of enhancing the therapeutic efficacy in immunosuppressed TB patients as the antibody is independent of Tregs regulation as well as capable of exhibiting ADCC mechanism. In a similar context, a TCR-like antibody would be equally beneficial in reducing the risk of latent TB reactivation during/post-COVID-19 which is associated with the depletion and exhaustion of T cells.

The functions of TCR-like antibodies are not solely confined to the basic antibody effector mechanisms and can be further manipulated to enhance their effectiveness. One significant strategy is to incorporate the TCR-like antibody as antibody-based immunotoxins. In the aspect of tuberculosis, the TCR-like antibody-based immunotoxins would contribute to the elimination of TB-infected cells without affecting the normal cells (178). Other modifications include antibody fusion molecules and combinational therapy which will improve the overall treatment efficiency of many diseases including TB, infection, cancer, infectious diseases and many more (179). So far, there are several successful preliminary reports on the generation of TCR-like antibodies against *Mtb* using a human single domain antibody phage display library (180–182). On the other hand, the ability of TCR-like antibodies to mediate ADCC was successfully demonstrated in both *in vitro* and *in vivo* cancer studies (183). Although the concept is relatively new and there is a crucial need for further investigation and validation of the concept in a large setting before it can be incorporated as immunotherapy, these preclinical data has shed some light on the concept of TCR-like antibody and led a positive direction for future investigations.

A key element that differentiates TCR-like antibody from a typical antibody and serves as the basis of the antibody generation is the ability to detect antigen presentation on the MHC molecule (Human leukocyte antigen (HLA) in humans). Sadly, MHC class I expressions are found to be downregulated by *Mtb* (184) and even the SARS-COV-2 virus (185). However, it is important to take note that most studies have reported reduced MHC expression and there are no strong evidences of a complete loss of MHC expression in TB patients to date. As such, several strategies can be developed to improve MHC presentation in TB patients and consequently enhance the therapeutic efficacy of TCR-like antibodies. It would be ideal to design the improvement strategy based on the factors or mechanisms evoked by *Mtb* to downregulate MHC class I expression (186–190). For example, the proline-proline-glutamic acid (PPE) 38 protein of the *Mtb* is an ideal candidate to target not only for the improvement of MHC presentation but also as an antigenic candidate for the generation of a therapeutic antibody as it has been proven to inhibit MHC class I expression (*in vivo* study) (184). Similarly, secreted *Mtb* antigen Ag85B which has been associated with the alteration of MHC presentation is another potential candidate to consider for enhancing MHC presentation (191). Another challenge to overcome in the application TCR-like antibody is HLA specificity. The antibody’s specificity to a particular type of HLA indicates that only the individuals with the same HLA could benefit from the antibody. One way to overcome this limitation is to carefully select the HLAs based on the predominant distribution of HLA globally. HLA-A2 is known to be found in the majority of the world population while HLA-A11 and HLA-A24 are common in the Asian population (179). Previously, all three HLAs were selected in a TCR-like antibody study to ensure the findings were applicable for a wide range of population (180, 181). Overall, these are merely hiccups that can be overcome to ensure the successful application of TCR-like antibodies in TB therapeutics.

**Figure 2:**
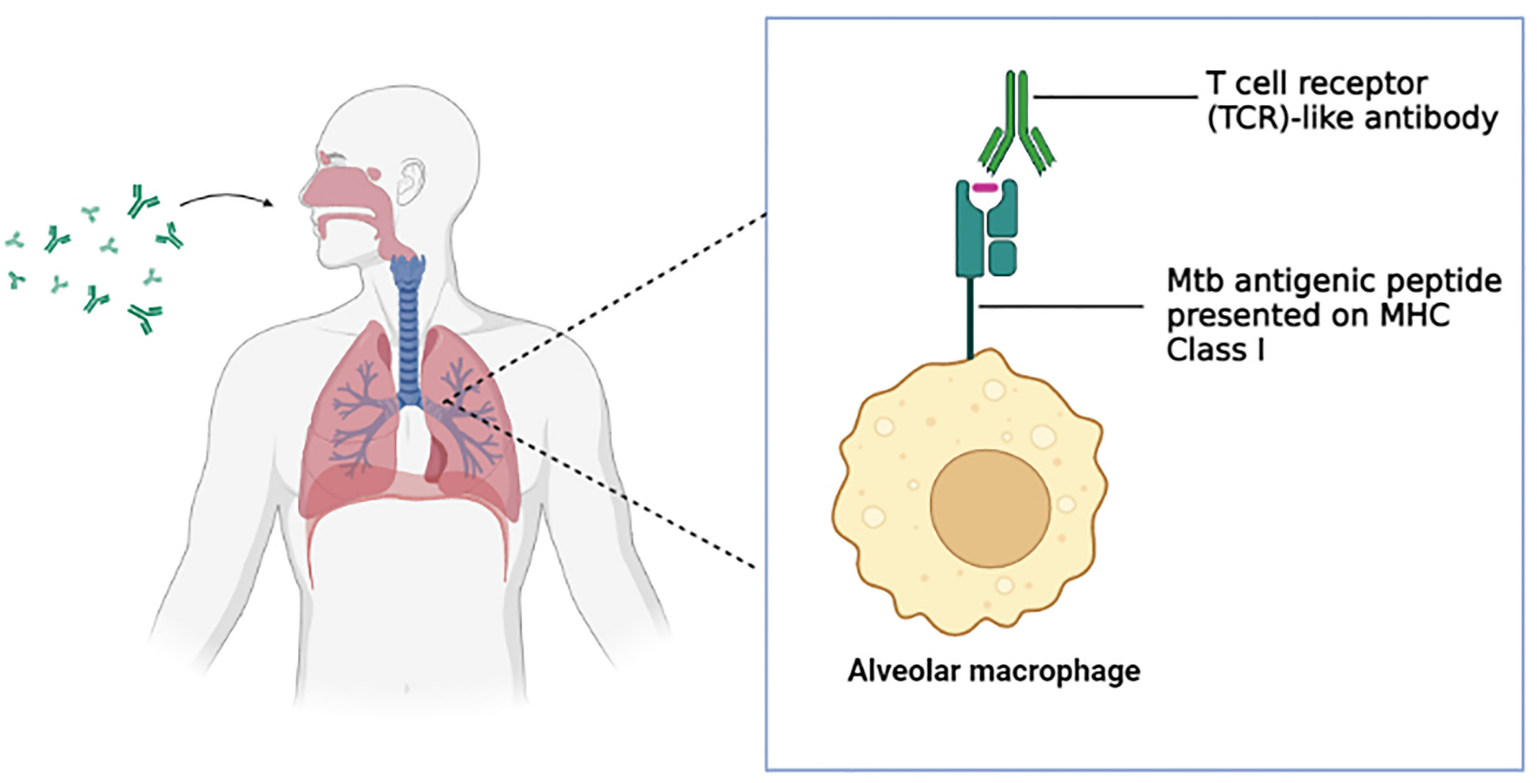
T cell receptor (TCR)-like antibody *via* inhalation delivery method. The inhaled antibody is capable of binding to the *Mtb* antigenic peptide presented on the MHC class I complex of the alveolar macrophages and subsequently mediate a wide range of immune defense mechanism. (Created in BioRender.com).

One major obstacle faced in monoclonal antibody therapy for respiratory diseases is the efficient delivery of the monoclonal antibody to the target organ (lung) (192). This is precisely true in tuberculosis as the delicate structure of the alveoli designed along with the acquired mobility (inflation and deflation) for gas exchange hinders the antibodies from reaching the alveoli (39). Traditionally, an intravenous injection was used for antibody delivery although the method lacked in the aspects of a requirement of high dosage, a limited amount of the antibodies were successfully delivered to the lung and potential side effects (192). Inhalation delivery of antibodies surpassed the obstacles of intravenous injection and has a lot of potentials to be successful (193–195). The application of the generated TCR-like antibodies in the inhalation delivery method (**Figure 2**) serves as a promising platform for the efficient therapeutics of tuberculosis. The compact format of the TCR-like domain antibody fragment enables longer retention of the antibody in the lung, enhance tissue penetration and binding to cryptic epitopes, as well as cost-effective production *via* bacterial expression system (196) As such, effective immunotherapy for tuberculosis can be achieved.

## Conclusion

The severity of COVID-19 has paused the entire world and reshaped the life of humankind on a grand scale. However, focusing solely on the pandemic has led to many negative implications. The TB/COVID-19 syndemic alone has significantly impacted the general public health, especially TB patients. Consequently, the aim to eradicate TB by 2030 is now further delayed due to the shift of focus to COVID-19. Moving forward, the valuable lessons learnt and experiences gained from the pandemic enable us to be prepared to handle any future syndemic/pandemic and pave new strategies/platforms to target TB. Notably, the dysfunctionality of T cells that has been associated with the implications of TB/COVID-19 syndemic serves as an ideal target for future research exploring other alternative therapeutic approaches, including therapeutic antibodies for TB. In addition, the advancement in technology, including phage display technology, enables the development of novel antibody classes such as T cell receptor (TCR)-like antibody that is primarily independent of T cell mechanisms with bright potentials to enhance the therapeutic efficacy of TB in both in an independent and syndemic scenario.

## Author Contributions

Conceptualization, SD and GT. Writing—original draft preparation, SD. Writing—review and editing, SD, GT, VB, CL, NA, and FN. Supervision, GT and FN. Funding acquisition, GT and FN. All authors contributed to the article and approved the submitted version.

## Funding

Acknowledgement to “Ministry of Higher Education Malaysia for Fundamental Research Grant Scheme with Project Code: FRGS/1/2020/STG01/USM/02/12”, “Ministry of Higher Education Malaysia for Higher Institution Centre of Excellence (HICoE: 311/CIPPM/4401005)” and "Medical Faculty of Universiti Kebangsaan Malaysia research grants (FF-2020-327)".

## Conflict of Interest

The authors declare that the research was conducted in the absence of any commercial or financial relationships that could be construed as a potential conflict of interest.

## Publisher’s Note

All claims expressed in this article are solely those of the authors and do not necessarily represent those of their affiliated organizations, or those of the publisher, the editors and the reviewers. Any product that may be evaluated in this article, or claim that may be made by its manufacturer, is not guaranteed or endorsed by the publisher.
